# Feeding recycled food waste improved feed efficiency in laying hens from 24 to 43 weeks of age

**DOI:** 10.1038/s41598-023-34878-2

**Published:** 2023-05-22

**Authors:** Hiep T. Dao, Nishchal K. Sharma, Robert A. Swick, Amy F. Moss

**Affiliations:** 1 School of Environmental and Rural Science, Faculty of Science, Agriculture, Business and Law, University of New England, Armidale, NSW 2351 Australia; 2 Faculty of Animal Science, Vietnam National University of Agriculture, Trau Quy Town, Gia Lam District, Hanoi, 100000 Vietnam

**Keywords:** Zoology, Environmental sciences

## Abstract

There is renewed interest in utilizing food waste as animal feed due to its potential benefits in reducing feed cost and environmental impact while improving global food security. This study was conducted to examine the efficacy of recycled food waste-based feed for laying hen performance, egg quality, and nutrient digestibility. Hy-Line Brown hens (*n* = 150) were randomly distributed to three dietary treatments with 50 replicate cages of a single bird per treatment from 24 to 43 weeks of age. The treatments were: a standard/control feed based on wheat, sorghum, and soybean meal; a recycled food waste based-feed; and a 50:50 blend of control and food waste based-feed. Hens offered the food waste-based diets had similar egg weight, hen day egg production, and egg mass, but lower feed intake and higher feed efficiency, compared to those fed the control diets (*P* < 0.001). Hens fed the food waste diets exhibited lower shell breaking strength and shell thickness at week 34, and higher yolk color score and higher fat digestibility compared to the control treatment at week 43 (*P* < 0.001). Thus, feeding the recycled food waste based-feed maintained egg production while improving feed efficiency compared to the control feed.

## Introduction

It is estimated that about one-third of all food produced globally is lost as waste, causing a loss of US$ 1 trillion annually^1^. In Australia, approximately 7.3 million tonnes of food is disposed in landfill per year, which costs more than US$ 14 billion to the Australian economy. This waste also contributes to more than 5% of Australia’s greenhouse gas emissions, leading to substantial environmental and economic losses^2^. As food is wasted, the costs associated with the production, processing, delivery, and selling of that food are also lost. Moreover, the global warming potential caused by 1 ton of food waste in landfill is more than 5 times higher than that of recycling food waste into dry animal feed^3^. Simultaneously, poultry feed occupies a major cost to producers and its price has increased due to rising prices of raw materials.

Previous studies have illustrated the possibility of producing feed from food waste that meets nutritional requirements for poultry, as well as hygiene and chemical safety standards^4,5^. A comprehensive review by Torok et al.^6^ concluded that food waste can be effectively and safely utilized in commercial production systems. Some processed food waste streams such as spent brewers grain, fish offal- spent brewers grain blend, and meat and bone meal may replace costly grains, oil, and protein meals in poultry diets thus reducing feed cost significantly^7,8^. Creating poultry feed from food waste is also expected to lower carbon and greenhouse gas emissions in the production of chicken meat by 35% and 25% respectively, and in eggs by 75% and 76% respectively^9^. Similarly, recycling food waste into pig feed may lead to better public health and environmental effects compared to other processing methods, such as anaerobic digestion and composting^10^. Therefore, there is great economic and environmental opportunity in the creation of poultry feed from food waste. While this concept is new to many countries, using food waste based-feed has been an ongoing practice for many years and is supported by local governments in Japan and South Korea^11,12^. It is estimated that approximately 40% and 46% of mixed food waste are recycled as livestock and poultry feed respectively in these countries^13^. Others including Taiwan and the US have already used processed food waste as animal feed^14^. This study aimed to investigate the efficacy of recycled food waste-based feed on laying performance, egg quality, and nutrient digestibility of laying hens by comparing a commercial diet with a food waste diet and a 50:50 blend of the two. It was hypothesized that laying hens would perform up to the breeder specifications when fed diets containing 100% food waste.

### Methods

All experimental procedures were approved by the University of New England Animal Ethics Committee (AEC20-042). This study was performed in accordance and full compliance with the approved guidelines and regulations. The study reported in this paper follows the recommendations in ARRIVE guidelines.

### Experimental design and diets

**Figure 1:**
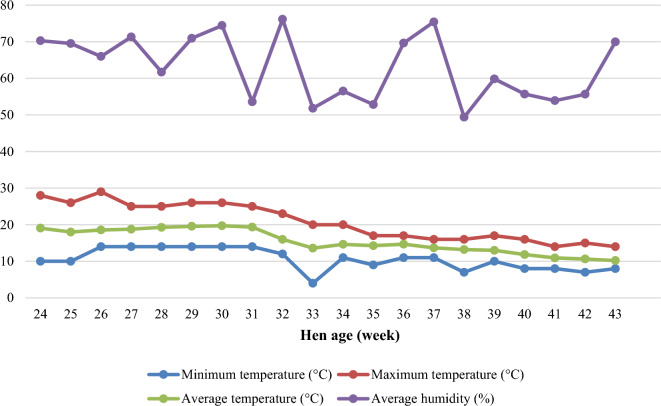
Temperature and relative humidity of the hen house from 24 to 43 weeks of age.

The study was implemented at the University of New England Laureldale Cage layer facility in Armidale, New South Wales, Australia. One hundred fifty Hy-Line Brown pullets were purchased from a commercial laying hen farm in Tamworth, New South Wales, Australia at 15 weeks of age. Birds were fed a pre-lay diet (2,800 kcal ME/kg, 16.7% crude protein, 2.6% calcium, 0.48% available phosphorus) from 15 to 19 weeks of age and a commercial layer diet from 19 to 22 weeks of age (2,750 kcal ME/kg, 16.5% crude protein, 3.6% calcium, 0.4% available phosphorus; Barastoc Premium Top Layer Mash, Ridley Corporation Ltd., Melbourne, Victoria, Australia). At 23 weeks of age, birds were weighed and randomly allocated to 3 dietary treatments: standard/control feed based on wheat, sorghum, and soybean meal; recycled food waste based-feed; and a 50:50 blend of control and food waste-based feed. There were 50 replicate hens per treatment, housed individually. The average starting hen weights were not different between the dietary treatments (*P* > 0.05). Experimental diets were gradually increased during a 10-day adaptation period and were then fed to birds from week 24. Feed intake (FI) from 15 to 22 weeks of age was employed to formulate the experimental diets according to Hy-Line Brown nutritional requirements^15^. The study was implemented over a 20 weeks period until the hens were at 43 weeks of age. Birds were housed individually in cages (30 cm width × 50 cm depth × 45 cm height) in a curtain-sided house. There were two nipple drinkers and one feeder per bird. Birds had free access to feed and water. A lighting program of 16 h light: 8 h dark was maintained throughout the study. Temperature and relative humidity in the hen shed were recorded daily throughout the study but were not controlled. The average hen house temperature and relative humidity by weeks are shown in Fig. 1.

All diets met the minimum nutrient requirements of Hy-Line Brown hens (2,700 kcal ME/kg, 14.0% crude protein, 4.0% calcium, 0.4% available phosphorus) according to Hy-Line Brown nutritional recommendations for the laying period^15^. Diets were offered as mash and contained two feeding periods: 24 to 37 weeks and 38 to 43 weeks. Feedstuffs were analyzed for particle size distribution and nutrient content including dry matter (DM), gross energy (GE), crude protein (CP), amino acids (AA), crude fat, crude fiber, and mineral composition using standard procedures^16^ prior to diet formulation (Tables 1 and 2). The particle size distribution of the diets was measured by dried sieving using a shaker (Retsch AS 200 digit cA, Retsch GmbH, 42,781 Haan, Germany) provided with 8 sieves (4, 2.8, 2, 1.6, 1.25, 1, 0.5, and 0.25 mm screen). Metabolizable energy and total and digestible AA levels of wheat, sorghum, soybean meal, canola meal, and meat and bone meal used in the control diet were obtained from near-infra red reflectance spectroscopy (Foss NIR 6500, Denmark) and standardized with Evonik AMINONIR Advanced calibration. The metabolizable energy and digestible AA levels of the food waste materials were estimated at 65% based on previous reports^17–19^. Dry matter, GE, CP, AA, crude fat, crude fiber, ash content, and mineral composition of mixed control and food waste-based diets were analyzed by standard methods^16^ to confirm the accuracy of the dietary composition. Food waste materials were collected from breweries, hospitals, nursing homes, bakeries, pubs and restaurants, abattoirs, fish processing facilities, and vegetable and fruit markets. After removing foreign objects, collected food waste was separated into general classifications including spent brewers grain, fish offal and spent brewers grain blend, hospital and nursing home meal, pub and restaurant meal, vegetable and fruit meal, meat and bone meal, bakery meal, and oyster shell meal. Each food waste stream was processed by Food Recycle Ltd. using their patented production process to create a granular powder (patent number 2018100266)^20^, which is then in a suitable form to feed to poultry. Then, waste streams were blended into a complete mash feed. Steam heating to exceed 100 °C for 30 min as described in Boyle^20^ was used during the food waste processing to ensure the inactivation of pathogenic and spoilage organisms. Minors such as crystalline AA (L-lysine HCl, L-threonine, D,L-methionine), xylanase, phytase, red and yellow pigments, antioxidant, and layer vitamin-mineral premix were added to all diets. The diet composition and nutrient content of dietary treatments are given in Tables 3, 4, 5, and 6. The analyzed nutrient content of the dietary treatments (Tables 4, 5, and 6) showed that the mixed diets met the minimum nutrient requirements of Hy-Line Brown hens according to the breed recommendation. Thus, the main feed formulation objective of this study was achieved. However, the nutrient composition of the control and recycled food waste-based diets were different. Of concern was the high sodium, phosphorus, and fat levels in the food waste-based diets. These nutrients were reduced as much as possible during feed formulation. However, it was not possible to produce 100% food waste-based diets with the same nutrients as the control diets. The objective of the study was to determine how laying hens would perform on 100% food waste-based diets. The protein, fat, sodium, and/or phosphorus levels of various food waste streams such as pub and restaurant meal, hospital and nursing home meal, fish offal meal, and meat and bone meal were high. Due to the nature of the food waste streams, previous studies might not attempt to make food waste-based diets isonitrogenous or isocaloric compared to the control diets^21^. Similar to this study, the protein and fat content in the food waste-based diet reported by Garnida et al.^22^ were also higher than the control diet.

**Table 1:** Analyzed nutrient values of food waste materials (as-is basis, %, otherwise as indicated). Values of all the amino acids presented were total amino acids (measured on an as-is basis). ^a^Fish offal and spent brewers grain blend was made by blending fish offal and spent brewers grain together (50% each in volume).

Nutrient	Spent brewers grain	Fish offal and spent brewers grain blend^a^	Pub and restaurant meal	Meat and bone meal	Bakery meal	Vegetable and fruit meal	Hospital and nursing home	Oyster shell meal
Dry matter	98.05	97.50	89.78	94.74	99.15	89.50	89.40	97.34
Crude protein	25.55	37.42	27.72	35.33	17.89	16.35	23.40	0.62
Gross energy, kcal/kg	5155	5414	4251	4009	4571	3298	4469	3728
Crude fat	9.83	24.81	23.28	21.74	5.60	2.95	19.60	0.10
Crude fiber	17.33	7.95	4.11	3.24	2.56	14.84	2.42	1.35
Acid detergent fiber	31.45	6.77	1.99	0.37	4.25	19.96	3.47	66.15
Neutral detergent fiber	66.54	24.29	11.91	11.90	9.01	21.08	12.91	5.64
Ash	3.49	12.80	24.85	32.82	3.37	15.84	20.70	94.52
Calcium	0.226	4.783	8.673	13.574	0.589	3.543	8.200	38.127
Total phosphorus	0.531	2.347	3.413	6.294	0.269	0.459	3.000	0.022
Sodium	0.016	0.324	2.293	0.594	0.674	0.890	–	0.477
Potassium	0.055	0.379	0.431	0.348	0.280	3.084	0.520	0.000
Lysine	1.106	2.043	1.714	1.823	0.519	0.521	1.300	0.056
Methionine	0.271	0.696	0.517	0.550	0.283	0.144	0.370	0.014
Threonine	0.980	1.414	1.033	1.086	0.556	0.494	–	–
Histidine	0.594	0.763	0.607	0.705	0.395	0.306	0.470	< 0.005
Arginine	1.250	2.266	1.733	2.489	0.735	0.726	1.500	0.045
Isoleucine	1.133	1.376	1.023	0.963	0.675	0.465	0.790	0.027
Leucine	2.010	2.311	1.883	1.961	1.198	0.714	1.600	0.046
Valine	1.484	1.701	1.321	1.404	0.782	0.690	–	–
Serine	1.104	1.509	1.072	1.283	0.811	0.587	–	–
Glycine	1.126	3.669	2.879	5.209	0.888	0.535	–	–
Aspartic acid	1.799	2.737	2.353	2.522	0.944	1.233	–	–
Glutamic acid	5.200	5.175	3.888	4.436	5.178	2.507	–	–
Alanine	1.272	2.443	1.925	2.716	0.707	0.731	–	–
Proline	2.505	2.657	1.980	3.195	1.773	0.680	–	–
Tyrosine	0.587	0.750	0.657	0.652	0.363	0.253	–	–
Phenylalanine	1.441	1.425	1.035	1.126	0.829	0.571	0.910	0.041

**Table 2:** Particle size distribution of feed ingredients.

Particle size (X, mm)	X ≥ 4	4 > X ≥ 2.8	2.8 > X ≥ 2	2 > X ≥ 1.6	1.6 > X ≥ 1.25	1.25 > X ≥ 1	1 > X ≥ 0.5	0.5 > X ≥ 0.25	X < 0.25
Bakery	13.79	55.86	6.02	4.01	4.05	3.34	8.80	3.37	0.76
Spent brewers grain	0.00	0.00	0.13	0.17	0.28	2.47	24.12	39.58	33.25
Vegetable and fruit meal	0.00	0.00	0.00	0.00	0.89	1.36	25.43	35.38	36.92
Recycled meat and bone meal	12.11	56.47	4.87	3.86	3.67	3.13	8.41	4.93	2.55
Pub and restaurant meal	5.80	53.69	3.28	3.39	4.56	4.02	9.97	15.29	0.00
Fish offal and spent brewers grain blend	3.21	51.92	2.22	2.08	3.42	4.14	28.60	4.43	0.00
Hospital and nursing home	6.92	53.67	2.88	3.24	2.71	2.62	27.24	0.72	0.00
Oyster shell meal	2.76	52.75	4.04	3.37	3.81	3.53	10.66	7.58	11.51
Sorghum	0.00	50.68	11.01	20.73	7.71	2.03	3.81	2.87	1.17
Wheat	0.00	50.09	6.91	13.89	11.41	4.57	6.50	3.55	3.10
Soybean meal	1.94	52.81	4.22	4.52	6.83	7.00	17.26	4.52	0.90
Canola meal	0.00	50.03	0.58	1.34	2.53	3.55	20.84	12.78	8.36
Common meat and bone meal	0.08	49.88	0.12	4.34	5.85	4.53	14.44	19.64	1.13
Limestone grit	0.13	61.66	14.40	9.07	7.54	4.57	2.49	0.11	0.02

**Table 3:** Diet composition for experimental treatments (as-is basis, %, otherwise as indicated). The diets were formulated using a feed formulation software (Concept 5, CFC Tech Services, Inc., USA). ^a^Control diet based on common feed ingredients to mimic commercial layer hen feed. ^b^Food waste diet based on recycled food waste materials. A 50:50 treatment was made by blending the control diet and food waste diet together (50% each in weight). ^c^Fish offal and spent brewers grain blend was made by blending fish offal and spent brewers grain together (50% each in volume). ^d^Econase XT, 25, AB Vista ^e^Quantum Blue 5G Layers, AB Vista**.** ^f^The composition of vitamin-mineral premix per kilogram diet was similar to that reported in to Dao et al.^21^. ^g^AMEn: N-corrected apparent metabolizable energy. ^h^SID: Standardized ileal digestibility. Digestible amino acid coefficients of conventional feed ingredients were determined by Near-Infra Red spectroscopy (Foss NIR 6500, Denmark) standardized with Evonik AMINONIR Advanced calibration.

Dietary treatment	24 to 37 week			38 to 43 week		
	Control^a^	Food waste^b^	50:50 blend	Control	Food waste	50:50 blend
Ingredients						
Wheat	40.75	0.00	20.38	46.30	0.00	23.15
Sorghum	20.00	0.00	10.00	20.00	0.00	10.00
Soybean meal	13.51	0.00	6.76	9.63	0.00	4.82
Canola meal	10.00	0.00	5.00	10.00	0.00	5.00
Commercial meat and bone meal	2.00	0.00	1.00	3.11	0.00	1.56
Canola oil	2.96	0.00	1.48	0.55	0.00	0.28
Limestone	9.60	0.00	4.80	9.80	0.00	4.90
Di-calcium phosphate	0.48	0.00	0.24	0.00	0.00	0.00
Salt	0.24	0.00	0.12	0.20	0.00	0.10
Vegetable and fruit meal	0.00	5.00	2.50	0.00	5.00	2.50
Spent brewers grain	0.00	34.90	17.45	0.00	28.19	14.10
Fish offal and spent brewers grain blend^c^	0.00	15.00	7.50	0.00	15.00	7.50
Hospital and nursing home meal	0.00	15.00	7.50	0.00	15.00	7.50
Pub and restaurant meal	0.00	3.11	1.56	0.00	2.81	1.41
Recycled meat and bone meal	0.00	8.30	4.15	0.00	8.00	4.00
Bakery meal	0.00	16.84	8.42	0.00	19.31	9.66
Oyster shell meal	0.00	1.28	0.64	0.00	6.16	3.08
Choline Cl 70%	0.061	0.268	0.165	0.066	0.268	0.167
L-lysine HCl	0.073	0.000	0.037	0.060	0.000	0.030
D,L-methionine	0.169	0.166	0.168	0.139	0.130	0.135
L-threonine	0.016	0.000	0.008	0.000	0.000	0.000
Xylanase^d^	0.005	0.005	0.005	0.005	0.005	0.005
Phytase^e^	0.006	0.006	0.006	0.006	0.006	0.006
Pigment jabiru red	0.004	0.004	0.004	0.004	0.004	0.004
Pigment jabiru yellow	0.003	0.003	0.003	0.003	0.003	0.003
Antioxidant	0.025	0.025	0.025	0.025	0.025	0.025
Vitamin-mineral premix^f^	0.100	0.100	0.100	0.100	0.100	0.100
Calculated composition						
AMEn^g^, kcal/kg	2800	2800	2800	2700	2700	2700
Crude protein	17.80	25.57	21.69	17.00	24.10	20.55
Crude fat	5.28	13.44	9.36	3.05	12.83	7.94
Crude fiber	2.78	8.91	5.85	2.79	7.87	5.33
SID^h^ arginine	0.945	0.962	0.954	0.872	0.913	0.893
SID lysine	0.780	0.808	0.794	0.700	0.761	0.731
SID methionine	0.420	0.445	0.433	0.380	0.398	0.389
SID cysteine	0.298	–	–	0.290	–	–
SID methionine + cysteine	0.719	0.670	0.695	0.671	0.600	0.636
SID tryptophan	0.213	0.193	0.203	0.198	0.183	0.191
SID histidine	0.378	–	–	0.352	–	–
SID phenylalanine	0.707	–	–	0.656	–	–
SID leucine	1.196	1.333	1.265	1.125	–	–
SID isoleucine	0.630	0.739	0.685	0.583	0.690	0.637
SID threonine	0.560	0.613	0.587	0.507	0.578	0.543
SID valine	0.733	0.962	0.848	0.691	0.894	0.793
Calcium	4.200	4.200	4.200	4.257	5.900	5.079
Available phosphorus	0.450	1.020	0.735	0.400	0.992	0.696
Sodium	0.180	0.450	0.315	0.170	0.480	0.325
Potassium	0.704	–	–	0.649	–	–
Chloride	0.222	–	–	0.201	–	–
Choline, mg/kg	1.400	1.400	1400	1.400	1.400	1400
Linoleic acid	1.582	–	–	1.000	–	–

**Table 4:** Analyzed nutrient values of experimental diets (as-is basis, %, otherwise as indicated). Values of all the amino acids presented were total amino acids (measured on an as-is basis). ^a^Control diet based on common feed ingredients to mimic commercial layer hen feed. ^b^Food waste diet based on recycled food waste materials. ^c^50:50 blend diet was made by blending the control diet and food waste diet together (50% each in weight).^d^Apparent metabolizable energy was measured by the total collection method or calculated from the gross energy and energy digestibility of the diets.

Dietary treatment	24 to 37 week			38 to 43 week		
	Control^a^	Food waste^b^	50:50 blend^c^	Control	Food waste	50:50 blend
Dry matter	91.16	93.00	91.80	91.02	91.24	91.00
Gross energy, kcal/kg	3717	4501	4001	3523	4175	3748
AME^d^, kcal/kg	2758	3245	2917	2815	3358	3126
Crude protein	17.93	22.93	20.12	17.40	19.60	17.96
Crude fat	4.38	9.57	7.35	5.19	6.76	6.06
Crude fiber	8.72	12.99	10.62	9.00	9.54	9.49
Ash	13.51	9.93	11.16	15.22	11.28	12.80
Calcium	4.99	3.13	3.96	5.71	4.04	5.38
Total phosphorus	0.56	1.31	0.91	0.58	0.92	0.78
Sodium	0.14	0.38	0.28	0.14	0.32	0.26
Potassium	0.74	0.41	0.53	0.66	0.43	0.58
Arginine	1.006	1.165	1.056	0.969	0.987	0.983
Lysine	0.867	0.935	0.911	0.829	0.786	0.805
Methionine	0.393	0.395	0.393	0.394	0.410	0.399
Histidine	0.452	0.458	0.453	0.425	0.402	0.410
Phenylalanine	0.823	1.018	0.882	0.764	0.827	0.825
Leucine	1.399	1.524	1.480	1.309	1.266	1.290
Isoleucine	0.725	0.849	0.732	0.675	0.695	0.677
Threonine	0.658	0.771	0.708	0.616	0.635	0.634
Valine	0.851	1.074	0.971	0.810	0.866	0.846
Glycine	0.851	1.632	1.057	0.966	1.376	0.969
Serine	0.808	0.915	0.829	0.769	0.763	0.764
Glutamic acid	3.641	4.151	3.814	3.487	3.595	3.499
Proline	1.232	1.906	1.472	1.257	1.590	1.319
Alanine	0.846	1.209	0.993	0.841	1.003	0.887
Tyrosine	0.451	0.514	0.487	0.412	0.469	0.456
Aspartic acid	1.388	1.548	1.435	1.261	1.295	1.269

**Table 5:** Analyzed free sugar and non-starch polysaccharide (NSP) content of experimental diets from weeks 24 to 37 (as-is basis, g/kg). ^a^Control diet based on common feed ingredients to mimic commercial layer hen feed. ^b^Food waste diet based on recycled food waste materials. ^c^50:50 blend diet was made by blending the control diet and food waste diet together (50% each in weight). ^d^SNSP: soluble NSP. ^e^INSP: insoluble NSP.

Nutrients	Control^a^				Food waste^b^				50:50 blend^c^			
	Free Sugars	SNSP^d^	INSP^e^	Total NSP	Free Sugars	SNSP	INSP	Total NSP	Free Sugars	SNSP	INSP	Total NSP
Rhamnose	0.00	0.00	0.00	0.00	0.00	0.16	0.00	0.16	0.00	0.06	0.00	0.06
Fucose	0.00	0.00	0.64	0.64	0.00	0.00	0.00	0.00	0.00	0.00	0.25	0.25
Ribose	0.00	0.44	0.00	0.44	0.00	0.19	0.00	0.19	0.00	0.28	0.00	0.28
Arabinose	0.53	3.52	18.25	21.77	0.81	3.28	36.42	39.70	0.60	3.31	25.41	28.72
Xylose	0.00	3.52	14.87	18.38	0.99	3.87	71.28	75.15	0.49	3.63	41.21	44.84
Mannose	4.65	1.13	1.33	2.45	3.15	1.34	1.76	3.10	4.57	1.21	1.68	2.89
Galactose	6.44	1.80	8.90	10.69	1.68	1.89	6.83	8.72	3.07	1.83	6.74	8.57
Glucose	18.24	1.55	22.99	24.54	16.77	1.75	16.96	18.72	17.35	1.62	19.21	20.83
Total	29.85	10.61	59.31	69.92	23.39	11.09	117.78	128.87	26.09	10.66	102.32	112.98
Starch (%)	35.66				14.49				25.14

**Table 6:** Analyzed free sugar and non-starch polysaccharide (NSP) content of experimental diets from weeks 38 to 43 (as-is basis, g/kg). ^a^Control diet based on common feed ingredients to mimic commercial layer hen feed. ^b^Food waste diet based on recycled food waste materials. ^c^50:50 blend diet was made by blending the control diet and food waste diet together (50% each in weight). ^d^SNSP: soluble NSP. ^e^INSP: insoluble NSP.

Nutrients	Control^a^				Food waste^b^				50:50 blend^c^			
	Free Sugars	SNSP^d^	INSP^e^	Total NSP	Free Sugars	SNSP	INSP	Total NSP	Free Sugars	SNSP	INSP	Total NSP
Rhamnose	0.00	0.00	0.00	0.00	0.00	0.13	0.00	0.13	0.00	0.05	0.00	0.05
Fucose	0.00	0.00	0.58	0.58	0.00	0.00	0.00	0.00	0.00	0.00	0.22	0.22
Ribose	0.00	0.42	0.00	0.42	0.00	0.19	0.00	0.19	0.00	0.26	0.00	0.26
Arabinose	0.44	3.62	17.76	21.38	0.46	2.77	17.69	20.46	0.42	2.91	17.69	20.60
Xylose	0.00	3.37	14.74	18.11	0.51	3.08	37.59	40.67	0.20	3.15	24.67	27.81
Mannose	3.88	1.25	1.33	2.59	3.73	2.05	1.79	3.84	3.81	1.44	1.82	3.25
Galactose	5.95	1.74	7.48	9.23	1.91	1.41	3.58	5.00	2.89	1.53	4.43	5.96
Glucose	17.65	1.59	23.71	25.30	19.56	6.20	39.06	45.26	18.38	3.74	34.09	37.83
Total	27.93	10.65	58.14	68.78	26.17	14.12	88.64	102.76	25.71	11.77	75.65	87.43
Starch (%)	37.42				15.33				26.29			

### Data collection

Egg weight, hen day egg production, and egg mass were recorded daily. Feed consumption was recorded weekly. The FCR was calculated by dividing feed intake by egg mass. Mortality rate was recorded daily throughout the study. Individual hen weight was recorded every fifth week beginning on week 24. At weeks 34 and 43, fresh, clean, and normal-shape eggs from all hens were collected for egg quality measurements. At week 43, ten hens per treatment with body weights close to the average body weight of the treatment were chosen for measurements of DM, GE, CP, crude fat digestibility, apparent metabolizable energy (AME), apparent metabolizable to gross energy ratio (AME:GE), and N-corrected AME (AMEn) using the total excreta collection method according to Dao et al.^23^.

### Egg quality measurement

Eggshell reflectivity was measured by the TSS QCE-QCM equipment (Technical Services and Supplies, Dunnington, York, UK). Egg length and width were measured by a digital caliper. The egg shape index was calculated as a ratio of egg width to egg length. Eggshell breaking strength, shell thickness, albumen height, Haugh unit, yolk color, yolk height, yolk diameter, and yolk index were measured by a digital egg tester (DET6500, Nabel Co., Ltd, Kyoto, Japan). The egg yolk was collected on filter paper (CAT No. 1541–090, Whatman, Buckinghamshire HP7 9NA, UK) and weighed. Eggshell was rinsed, dried thoroughly, and weighed. The albumen weight was calculated by subtracting the weights of egg yolk and eggshell from the total egg weight. Then egg proportion was calculated by dividing the weight of each egg component by the intact egg weight.

### Nutrient digestibility

Excreta samples collected at 43 weeks of age were freeze-dried (Christ Alpha 1–4 LDplus, Osterode am Harz, Germany) and milled to pass through a 0.5 mm screen. Gross energy and protein content of the feed and excreta was determined using a Parr adiabatic oxygen bomb calorimeter (Parr Instrument Co., Moline, IL, US) and a nitrogen analyzer (LECO Corporation, St Joseph, MI, US), respectively. Crude fat of the feed and excreta was measured using Soxhlet method^24^ adapted as outlined by Holman et al.^25^. Apparent DM, GE, CP, and crude fat digestibility were calculated following equations described by Dao et al.^23^. Apparent metabolizable energy, AME:GE, and AMEn were calculated following equations described by Moss et al.^26^. All data were calculated on a DM basis.

### Data analysis

Data was analyzed by one-way ANOVA using R Commander (version 3.3.1, R Foundation for Statistical Computing, Vienna, Austria). Tukey's post hoc test was employed to identify pairwise differences between the treatments from significant ANOVA results (*P* ≤ 0.05).

## Results

### Environmental condition, analyzed dietary nutrient composition and mortality rate

The temperature and relative humidity inside the hen shed during the study are shown in Fig. 1. The average indoor temperature was 15.2 °C (ranging from 10.0 to 19.7 °C) while the average relative humidity was 63.4% (ranging from 49.4% to 76.1%) during the experimental period. The maximum daily temperature ranged from 13.0 to 29.0 °C (average 20.4 °C) while the minimum daily temperature ranged from 4.0 to 14.0 °C (average 10.4 °C).

The chemical composition of various waste streams is given in Table 1. The fish offal and spent brewers grain blend, pub and restaurant meal, and meat and bone meal waste contained high levels of CP, crude fat, and total phosphorus. The sodium content of the pub and restaurant meal was 2.29% being high relative to the requirement. Whereas, spent brewers grain and vegetable and fruit waste contained high fiber levels (17.3% and 14.8% respectively, Table 1). The final diets formulated with waste streams met the formulation objectives in terms of meeting the nutritional requirements of Hy-Line Brown laying hens. The analyzed nutrients of the control diet were similar to the calculated values. In the food waste diets, the analyzed CP, crude fat, calcium, and sodium levels were lower, while crude fiber level was higher than the calculated values (Tables 3 and 4). Nevertheless, it is notable that when formulated to meet the minimum nutrient requirements of the breed, food waste-based diets contained higher concentrations of CP, crude fat, crude fiber, total phosphorus, and sodium compared to the control diet. Additionally, the analyzed free sugars were lower and total non-starch polysaccharide was higher in the food waste diets compared to the control diets as shown in Tables 5 and 6. As the study progressed, and new batches of food waste were utilized, closer nutritional levels between the control and food waste diets were observed in the second period of the study from weeks 38 to 43 compared to the initial period (weeks 24 to 37). The particle size distribution test showed that certain amounts of over-size particles (≥ 4 mm) were still observed in bakery meal, recycled meat and bone meal, pub and restaurant meal, fish offal and spent brewers grain blend, hospital and nursing home meal, and oyster shell meal (Table 2). Whereas, high percentages of fine particles (≤ 0.5 mm) were detected in spent brewers grain (72.8%) and vegetable and fruit meal (72.3%, Table 2).

Over the entire study, birds in all dietary treatments were visibly healthy. The mortality rates of the control, food waste, and 50:50 blend treatments from 24 to 43 weeks of age were 0%, 0%, and 2%, respectively. There was only one mortality recorded in the 50:50 blend treatment and the mortality was not related to dietary treatment.

### Hen weight and laying performance

Hen weights and weight gain from 24 to 43 weeks of age are given in Table 7. Lower body weight was observed in hens offered the food waste-based diets compared to those offered the control diets at weeks 29 and 39 (*P* < 0.05, Table 7). Hen weight in the 50:50 blend treatment was intermediate between the control and food waste treatment (Table 7). Hens offered the food waste-based diets had lower weight gain compared to those fed the 50:50 blend diets over the entire study from 24 to 43 weeks (*P* < 0.01) and specifically from weeks 24 to 29 (*P* < 0.001) and 34 to 39 (*P* < 0.05, Table 7). Also, lower weight gains were observed in hens offered the food waste based-diets compared to those fed the control diets from weeks 24 to 29 (*P* < 0.001) and 39 to 43 (*P* < 0.001, Table 7).

**Table 7:** Hen weight from weeks 24 to 43. ^a,b^Means within rows not sharing a common suffix are significantly different at the 5% level of probability.

Variable	Control	Food waste	50:50 blend	SEM	*P* value
Hen weight, g					
Week 24	1959	1922	1926	9.71	0.234
Week 29	2097^b^	2019^a^	2086^ab^	12.56	0.023
Week 34	2215	2141	2184	13.61	0.086
Week 39	2278^b^	2185^a^	2269^ab^	15.56	0.025
Week 43	2285	2227	2296	15.48	0.148
Weight change, g					
Weeks 24–29	138^b^	96.9^a^	161^b^	5.78	< 0.001
Weeks 29–34	118	123	98.1	4.45	0.169
Weeks 34–39	63.6^ab^	43.6^a^	84.2^b^	5.64	0.013
Weeks 39–43	6.80^a^	42.2^b^	27.7^ab^	4.39	< 0.001
Weeks 24–34	256	219	259	7.22	0.064
Weeks 34–43	70.4^a^	85.8^ab^	111.9^b^	6.67	0.031
Weeks 24–43	326^ab^	305^a^	372^b^	9.03	0.008

**Figure 2:**
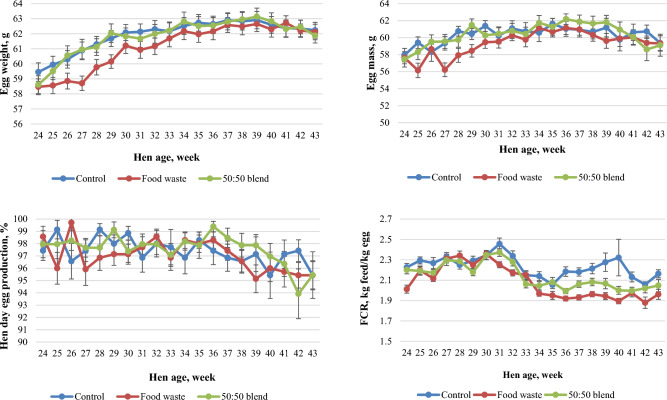
Egg weight, egg mass, hen day egg production, and feed to gain ratio (FCR) of hens fed the dietary treatments from 24 to 43 weeks of age. The dot points represent means and error bars present standard errors of the means.

The laying performance of dietary treatments from weeks 24 to 43 is given in Table 8 and Fig. 2. Hens offered the food waste-based diets had similar egg weight, hen day egg production, and egg mass, but lower feed intake (*P* < 0.001) resulting in a lower FCR (*P* < 0.001) compared to those fed the control diets from 24 to 43 weeks of age (Table 8). Specifically, hens fed the food waste diets had approximately 15 points lower FCR compared to those fed the control diets from 24 to 43 weeks of age. The 50:50 blend treatment had an intermediary response over weeks 24 to 43 (Table 8). Similar findings were observed in laying performance from weeks 24 to 33 and 34 to 43 (Table 8).

**Table 8:** Laying performance of hens fed dietary treatments from weeks 24 to 43. ^a–c^Means within rows not sharing a common suffix are significantly different at the 5% level of probability.

Hen age, week	Variable	Control	Food waste	50:50 blend	SEM	*P* value
24 to 33	Egg weight, g	61.2	60.0	61.1	0.30	0.172
	Hen day egg production,%	97.9	97.4	97.9	0.29	0.193
	Egg mass, g/day	60.0	58.4	59.8	0.34	0.133
	Feed intake, g/day	136^b^	129^a^	133^ab^	0.80	0.003
	FCR, kg feed/kg egg	2.284	2.205	2.229	0.015	0.082
34 to 43	Egg weight, g	62.6	62.3	62.6	0.29	0.902
	Hen day egg production,%	96.9	96.6	97.2	0.37	0.235
	Egg mass, g/day	60.7	60.2	60.9	0.38	0.748
	Feed intake, g/day	130^c^	116^a^	123^b^	0.94	< 0.001
	FCR, kg feed/kg egg	2.149^c^	1.931^a^	2.028^b^	0.017	< 0.001
24 to 43	Egg weight, g	61.9	61.2	61.9	0.29	0.468
	Hen day egg production,%	97.4	97.0	97.6	0.30	0.110
	Egg mass, g/day	60.3	59.3	60.4	0.35	0.313
	Feed intake, g/day	133^c^	122^a^	128^b^	0.81	< 0.001
	FCR, kg feed/kg egg	2.216^b^	2.068^a^	2.127^a^	0.015	< 0.001

### Egg quality

The egg quality of hens fed the dietary treatments at weeks 34 and 43 is given in Tables 9 and 10, respectively. Hens offered the food waste based-diets exhibited lower shell breaking strength (*P* < 0.001), shell thickness (*P* < 0.001), shell weight (*P* < 0.001), and shell proportion (*P* < 0.001) compared to the control and 50:50 blend treatments at week 34 (Table 9). However, all other egg quality parameters were not significantly different between the dietary treatments at week 34 (Table 9). At week 43, higher yolk color score was observed in hens offered the food waste-based diets compared to those fed the control and 50:50 blend diets (*P* < 0.001), but all other parameters, including shell measurements, were not significantly different between the dietary treatments (Table 10).

**Table 9:** Egg quality of hens fed dietary treatments at week 34. ^a–c^Means within rows not sharing a common suffix are significantly different at the 5% level of probability.

Variable	Control	Food waste	50:50 blend	SEM	*P* value
External egg quality					
Shell breaking strength (Kgf)	4.69^b^	3.98^a^	4.55^b^	0.07	< 0.001
Shell thickness (mm)	0.379^b^	0.331^a^	0.368^b^	0.004	< 0.001
Egg length (mm)	56.8	56.9	57.0	0.16	0.900
Egg width (mm)	44.3	44.3	44.2	0.12	0.254
Egg shape index	0.779	0.780	0.777	0.002	0.212
Reflectivity (%)	22.5	22.3	22.6	0.32	0.867
Internal egg quality					
Albumen height (mm)	8.69	9.39	9.00	0.31	0.352
Yolk colour	11.9	12.1	11.8	0.10	0.490
Haugh unit	88.9	91.5	91.5	1.57	0.387
Yolk height (mm)	22.3	22.5	22.7	0.10	0.355
Yolk diameter (mm)	41.9	42.3	42.8	0.33	0.463
Yolk index	0.532	0.533	0.533	0.004	0.995
Egg proportion					
Albumen weight (g)	41.13	41.14	40.35	0.30	0.473
Yolk weight (g)	16.28	16.46	16.44	0.11	0.783
Shell weight (g)	6.07^c^	5.43^a^	5.83^b^	0.04	< 0.001
Albumen (%)	64.70	65.23	64.40	0.18	0.137
Yolk (%)	25.70	26.14	26.29	0.15	0.270
Shell (%)	9.63^c^	8.62^a^	9.32^b^	0.06	< 0.001

**Table 10:** Egg quality of hens fed dietary treatments at week 43. ^a, b^Means within rows not sharing a common suffix are significantly different at the 5% level of probability.

Variable	Control	Food waste	50:50 blend	SEM	*P* value
External egg quality					
Shell breaking strength (Kgf)	4.41	4.43	4.37	0.07	0.933
Shell thickness (mm)	0.396	0.385	0.398	0.004	0.167
Egg length (mm)	56.7	56.8	57.0	0.16	0.720
Egg width (mm)	43.5	43.7	43.6	0.10	0.865
Egg shape index	0.769	0.77	0.766	0.002	0.747
Reflectivity (%)	25.0	24.9	25.5	0.31	0.681
Internal egg quality					
Albumen height (mm)	8.59	8.89	8.61	0.19	0.765
Yolk colour	12.5^a^	13.5^b^	12.9^a^	0.10	< 0.001
Haugh unit	90.1	92.3	90.8	1.08	0.874
Yolk height (mm)	22.1	22.2	22.3	0.08	0.742
Yolk diameter (mm)	41.4	41.8	43.6	0.60	0.491
Yolk index	0.537	0.534	0.527	0.005	0.738
Egg proportion					
Albumen weight (g)	40.19	40.10	40.14	0.30	0.993
Yolk weight (g)	16.29	16.81	16.36	0.11	0.109
Shell weight (g)	6.14	6.02	6.06	0.04	0.405
Albumen (%)	64.09	63.66	64.12	0.18	0.514
Yolk (%)	26.09	26.76	26.18	0.17	0.207
Shell (%)	9.82	9.57	9.71	0.05	0.162

### Excreta moisture and nutrient digestibility

The excreta moisture and nutrient digestibility of the dietary treatments at week 43 are shown in Table 11. Hens offered the food waste based-diets had higher excreta moisture than hens offered the control diets (*P* < 0.01, Table 11). Hens offered the food waste based-diets had a lower retained DM (*P* < 0.01) and digestibility (*P* < 0.05) compared to those fed the control diets at week 43 (Table 11). Hens fed the 50:50 blend diets exhibited a lower DM intake (*P* < 0.05) and retained DM (*P* < 0.01), but similar DM digestibility compared to those fed the control diets at week 43 (Table 11). Hens offered the food waste based-diets tended to have a higher energy consumption (*P* = 0.056) but lower energy digestibility (*P* = 0.056), and thus had a higher energy excretion (*P* < 0.01) compared to those fed the control and 50:50 blend treatments (Table 11). Higher AME and AMEn were observed in hens fed the food waste diets compared to the control diets (*P* < 0.001, Table 11). Hens offered the food waste based-diets had a higher protein intake (*P* < 0.05) and tended to have higher retained protein (*P* = 0.066) compared to those fed the 50:50 blend diets (Table 11). Noticeably, hens offered the food waste based-diets had a higher fat intake, retention, and digestibility compared to those offered the control diets (*P* < 0.001, Table 11). Hens fed the 50:50 blend diets showed an intermediary response (*P* < 0.001, Table 11).

**Table 11:** Excreta moisture and nutrient digestibility by total collection method at week 43. ^a-c^Means within rows not sharing a common suffix are significantly different at the 5% level of probability. ^d^AME: Apparent metabolizable energy. ^e^AME:GE: Apparent metabolizable energy to gross energy ratio. ^f^AMEn: N-corrected apparent metabolizable energy.

Variable	Control	Food waste	50:50 blend	SEM	*P* value
Excreta moisture, %	75.4^a^	78.2^b^	76.3^ab^	0.39	0.005
Dry matter digestibility					
Dry matter intake, g/day	118^b^	107^ab^	103^a^	2.48	0.022
Dry matter excreted, g/day	36.3	35.8	32.8	0.87	0.200
Dry matter retained, g/day	82.1^b^	71.0^a^	70.0^a^	1.87	0.009
Dry matter apparent digestibility, %	69.3^b^	66.6^a^	68.0^ab^	0.47	0.049
Energy digestibility					
Energy intake, kcal/day	449	497	441	10.31	0.056
Energy excreted, kcal/day	116^a^	139^b^	119^a^	3.38	0.007
Energy retained, kcal/day	333	359	321	7.66	0.127
Energy apparent digestibility, %	74.2	72.1	72.9	0.38	0.064
AME^d^ (kcal/kg)	2815^a^	3358^c^	3126^b^	43.65	< 0.001
AME:GE^e^	0.819	0.797	0.805	0.004	0.093
AMEn^f^ (kcal/kg)	2811^a^	3354^c^	3122^b^	43.63	< 0.001
Protein digestibility					
Protein intake, g/day	23.4^ab^	24.5^b^	21.4^a^	0.51	0.043
Protein excreted, g/day	12.9	12.9	11.9	0.25	0.140
Protein retained, g/day	10.5	11.6	9.58	0.36	0.066
Protein apparent digestibility, %	44.8	47.3	44.1	0.79	0.240
Fat digestibility					
Fat intake, g/day	6.77^a^	16.2^c^	10.2^b^	0.76	< 0.001
Fat excreted, g/day	2.23^a^	2.97^b^	2.42^a^	0.10	0.006
Fat retained, g/day	4.54^a^	13.2^c^	7.76^b^	0.69	< 0.001
Fat apparent digestibility, %	67.2^a^	81.7^c^	76.2^b^	1.35	< 0.001

## Discussion

The results of the current study demonstrated that feeding food waste based-diets could generate a higher feed efficiency in laying hens compared to when they are offered a standard diet based on wheat, sorghum, and soybean meal. The higher energy, fat, fiber, and protein levels in the food waste based-diets are likely reasons that hens consumed less feed and were more efficient in converting feed to egg mass than the control treatment. It is widely accepted that feed intake decreases as the dietary energy and/or fat level increases^27,28^. Furthermore, the high fat and protein content of food waste may result in a more efficient metabolism, possibly due to the higher net energy to AMEn ratio^29^. This is supported by the fat and protein digestibility results of the current study. Furthermore, as the fat source of the food waste based-diets mainly originated from fish offal and spent brewers grain blend, pub and restaurant meal, meat and bone meal, and hospital and nursing home meal, the fat contained in these waste streams might be more digestible than the canola oil used in the commercial feed counterparts. Fat digestibility of vegetable oils is often higher than animal oils for any single oil; however, balanced and combined oils may lead to a higher fat digestibility than single oils^30^. Others reported higher feed efficiency in laying hens fed lard (1.5%) compared to those fed soybean oil (1.5%)^31^. Further studies on the fatty acid profile of the food waste diets are warranted to determine the mechanism under the higher fat digestibility in hens offered the food waste diets compared to the controls. Meanwhile, the lower DM and energy digestibility in hens fed food waste based-diets compared to the control diets might be attributed to the higher fiber, higher total non-starch polysaccharides, and lower free sugar levels of food waste and the undesirable particle size within the food waste diet compared to the control. A diet high in fiber, non-starch polysaccharides, or one which contains undesirable particle sizes has been reported to reduce DM and energy digestibility^15,32–35^. Sourcing more waste stream options, better control of particle size during food waste processing, and optimizing a cocktail of enzymes may allow higher DM and energy digestibility in the food waste diets and minimize excess undigested nutrients in the excreta.

Hens offered food waste-based diets were in general lighter than those offered the control diets in the current study. However, as the hen weights in all dietary treatments were above the target weights for Hy-Line Brown hens^15^, the lower hen weight is likely advantageous. Overweight hens are a common industry issue. Previous reports have indicated that a fat/overweight hen is often associated with lower egg production and feed efficiency^36,37^. In the current study, hens on the control treatment consumed more feed than the food waste treatment, but instead of using it for production, the excess nutrients were likely deposited as fat and/or excess heat increment, explaining the extra weight and higher FCR of the control hens.

Most of the egg quality parameters were not different between the dietary treatments in the current study. However, hens fed the food waste based-feed had lower shell quality compared to those fed the control feed at week 34. The mineral level and particle size of the first batch of meat and bone meal and particle size of the first batch of oyster shell meal (weeks 24 to 37), which provided the majority of the dietary calcium, were highly variable and thus despite testing multiple samples, the calcium content and availability was underestimated. The lower feed intake in hens fed the food waste based-feed compared to those fed the control feed might also result in lower calcium consumption. Additionally, the high phosphorus level in the food waste based-feed might increase the calcium to phosphorus ratio compared to the control feed. These factors likely reduced the calcium/mineral intake resulting in lower shell quality in hens fed the food waste diets at week 34. After this became apparent, the diet was adjusted (weeks 38 to 43) to correct the calcium content, and subsequently the shell quality was quickly restored at week 43. Nutrient variability in the waste streams is one challenge with food waste based-feed as indicated by various studies^11,19^. This problem can be solved by blending large amounts of food waste at the same time to increase its consistency. Additionally, the selection of reliable waste sources such as large abattoirs or large bakery factories can also help with consistency^4,8^. Finally, creating an NIR calibration would allow rapid nutrient analysis and may reduce the impact of this challenge. Nevertheless, highly variable sources such as meat and bone meal should be tested more regularly or be avoided and replaced with a more stable source of calcium to avoid this issue on a commercial basis. Interestingly, hens fed the 50:50 blend diets could maintain similar shell breaking strength and shell thickness compared to those fed the control diets at week 34.

Hens fed the food waste-based diets had higher yolk color score compared to those fed the control diets at week 43. This is sensible as food waste diets may contain a higher xanthophyll and carotenoid level (likely from the fruit and vegetable waste), which are the main factors regulating yolk color^38,39^. In addition, the inclusion of fish offal meal (fish oil) in the food waste diets might also increase yolk color as previously observed by Mousavi et al.^40^. Other factors including dietary fat, calcium, vitamin A, and mycotoxin levels and anti-nutritional factors might also influence the yolk color score in hens fed the food waste based-diets in this study^41^. Darker yolks are preferred by consumers^39^; however, if the yolk color were too dark for consumer preference, this may be easily changed by slightly reducing the level of added pigment within the food waste-based diets.

It is demonstrated that food waste-based feed increased excreta moisture in the current study. This might increase the manure drying time and disposal costs and slightly increase issues with flies and odor; however, the difference was small and may have no noticeable impact. The increase in excreta moisture was likely due to the higher sodium and protein levels of food waste-based diets compared to the control diets. It has been reported that higher levels of these nutrients in the diets might increase water intake resulting in wetter litter^42–45^. High sodium content in the recycled food waste feed was also reported in previous investigations^4,19^. This could be an issue in areas of high humidity and may require lower use of the high salt waste streams or require the employment of desalination methods during processing.

Finally, the thermal comfort range for the metabolic and productive activity of laying hens is within 18 to 23.9 °C with the optimal temperature range from 19 to 22°C^46,47^. Cold temperatures (below 16 °C) have been reported to increase feed intake while decreasing nutrient digestibility, egg production, and feed efficiency in laying hens^48,49^. In the current study, the average egg mass of all dietary treatments was slightly greater (60.0 vs 57.2 g/day), but feed intake (128 vs 111 g/day) and thus FCR (2.137 vs 1.932) was higher than the Hy-Line Brown performance standards^15^. The low indoor temperature observed in the current study (15.2 °C) might explain the lower overall laying performance compared to the Hy-Line Brown standards. In addition, as the hen weights in all dietary treatments in the current study were higher than the Hy-Line Brown standards^15^, extra energy would be required for maintenance resulting in lower feed efficiency.

## Conclusion

Laying hen diets that sustained production were successfully formulated from food waste materials. Furthermore, hens fed the recycled food waste-based diet had higher feed efficiency compared to those fed the commercial control diet. The current study demonstrated that food waste not only has great potential as an alternative feed ingredient within poultry feed but can meet the nutrient requirements of laying hens. Further study to determine the nutrient digestibility, calcium and phosphate availability, and optimal particle size of the food waste streams and the economic efficiency (cost–benefit analysis) of feeding food waste based-diets is necessary to facilitate a precise feed formation and optimize the food waste based-diets for practical commercial use. Additionally, examining the effects of feeding food waste based-diets on the organoleptic properties of poultry products is crucial to facilitate the adoption of the poultry industry on the food waste based-feed.

## Data Availability

The data that support this study will be shared upon reasonable request to the corresponding author.
